# The Efficacy of Buccal Infiltration of 4% Articaine and PSA Injection of 2% Lidocaine on Anesthesia of Maxillary Second Molars

**DOI:** 10.22037/iej.v12i3.16464

**Published:** 2017

**Authors:** Ensiyeh Maljaei, Maryam Pourkazemi, Milad Ghanizadeh, Rana Ranjbar

**Affiliations:** a *Department of Pediatric Dentistry, Tabriz University of Medical Sciences, Tabriz, Iran; *; b *Department of Pediatric Dentistry, Tabriz University of Medical Sciences, Tabriz, Iran; *; c * Department of Oral and Maxillofacial Surgery, Dental School, Tabriz University of Medical Sciences, Tabriz, Iran; *; d *Dentist, Tabriz, Iran*

**Keywords:** Articaine, Buccal Infiltration Technique, Deciduous Second Molar, Lidocaine, Posterior Superior Alveolar Nerve Block

## Abstract

**Introduction:**

During the early mixed dentition period, the location of the deciduous maxillary second molar results in ineffectiveness of the infiltration technique in this area. In such cases, administration of posterior superior alveolar (PSA) nerve block is recommended; however, such a technique has some complications. The present study was undertaken to compare the effects of buccal infiltration of 4% Articaine and PSA technique with 2% Lidocaine on the success of anesthesia of maxillary deciduous second molars in 6 to 9-year old children.

**Methods and Materials:**

In the present double-blind randomized clinical trial, 56 children aged 6-9 years requiring vital pulp therapy of deciduous maxillary second molar were included. In group 1, 4% Articaine was injected using a buccal infiltration technique. In group 2, 2% Lidocaine was injected using the PSA nerve block technique. After 10 min, the caries was removed and access cavity preparation was instituted. The patients were asked to report the presence or absence of pain during the procedure. Therefore, the existence of pain was measured by the patient's self-report. Data were analyzed with descriptive statistical methods and the *chi*-squared test.

**Results:**

Pain was reported by 6 (21.4%) and 9 (32.1%) subjects in the Articaine and Lidocaine groups, respectively. *Chi*-squared test did not reveal any significant differences between the two groups (*P*=0.54).

**Conclusion:**

Under the limitations of the present study, there was no significant differences between the results of Articaine buccal infiltration and Lidocaine PSA technique, so Articaine buccal infiltration can be used as a substitute for the PSA technique.

## Introduction

Virtually all the dental procedures, including restorations, tooth extractions, pulpotomies, orthodontic procedures *etc.*, are associated with pain and discomfort; in this context, pain perception has become a major concern in moderns dentistry [1, 2]. Studies have shown that almost half of the patients refuse to attend dental offices due to fear of dental procedures [3]. Local anesthetic agents block the peripheral nerves and prevent the conduction of pain perception, making the patient and the dentist more comfortable [4]. Lidocaine is the most commonly used medication for the induction of local anesthesia. It is an amide anesthetic agent with a rapid action (45-90 sec) and a short duration of action (10-20 min); the effect increases if Lidocaine is used in association with adrenaline [5, 6].

The bone covering the deciduous maxillary first molar is thin; therefore, this tooth can be effectively anesthetized with the use of the infiltration technique. However, during the early mixed dentition period, the thick zygomatic process of the maxilla covers the buccal roots of the deciduous second molars and the permanent first molars in the maxilla, making the subperiosteal technique around the root apices of deciduous second molars ineffective. In such cases posterior superior alveolar (PSA) nerve block is recommended [7-9].

However, apart from the more difficult nature of this injection technique compared to the infiltration technique, it is associated with some complications, the most common of which is injury to the pterygoid venous plexus and formation of hematoma [10]. Some other complications of the PSA technique include trauma due to mastication, infection, trauma to sensory and motor nerves, trismus, blurred vision and in rare cases fracture of the needle in tissues [10-12].

Articaine is another anesthetic agent from the amide family with a half-life of 20 min. It is more soluble in lipids, compared to Lidocaine, due to the presence of a thiophene ring in its chemical structure so that it can easily penetrate into the lipid membrane of nerves [5, 13]. On the other hand, Articaine has strong affinity for proteins, making it's penetrate into bone possible [9, 14].

Srinivasan *et al.* [15] evaluated the effects of 4% Articaine and 2% Lidocaine (both containing 1:100,000 concentration of epinephrine) using the buccal infiltration technique of the upper first molars and first premolars and concluded that the success rate of Articaine was 100% in both areas but the success rates of Lidocaine in the first premolar and the first molar areas were 80% and 30%, respectively. Mittal *et al.* [16] showed that injection of Articaine in an infiltration technique in the maxillary deciduous molar area for the extraction of these teeth resulted in a more effective local anesthesia compared to Lidocaine. In a study by Yilmaz *et al.* [14], 162 children with pulpitis underwent injections of Prilocaine and Articaine using the maxillary infiltration or inferior alveolar nerve block technique. The results showed that the severity of pain at the time of removal of the coronal pulp in the Prilocaine group was 1.5 times higher. However, in a study by Arrow [17], although the inferior alveolar nerve block with Articaine was more successful than that with Lidocaine, no significant differences were observed in the mandibular buccal infiltration technique between the two anesthetic agents. Also Kanaa *et al.* [18], evaluated 100 patients with irreversible pulpitis of maxillary permanent teeth and concluded that Articaine and Lidocaine exerted similar effects on achieving local anesthesia.

Given the great thickness of the zygomatic process of the maxillary bone in the early mixed dentition period and the properties of Articaine in relation to its great ability to penetrate into bone, it is expected that infiltration of Articaine can be used as an alternative for posterior superior alveolar nerve block. 

No studies have been carried out to date to evaluate the use of Articaine for local anesthesia of maxillary deciduous molars needing vital pulp therapy and to compare it with the local anesthesia achieved with Lidocaine. The present study was undertaken to compare local anesthesia achieved with infiltration of 4% Articaine along with 1:100000 epinephrine with that achieved with the application of PSA nerve block with 2% Lidocaine with 1:80000 concentration of epinephrine in deciduous maxillary second molars requiring vital pulp therapy. Considering the great difficulty and possible complications of PSA nerve block, if favorable results are achieved, the use of infiltration anesthesia with the use of Articaine will be more favorable compared to the block technique.

## Materials and Methods

In the present double-blind randomized clinical trial, 56 male and female children aged 6 to 9 years, who were candidates for vital pulp therapy of deciduous maxillary second molars were selected and included in the study. All the ethical and the humanity considerations were considered and performed according to the Helsinki Declaration of 1975, as revised in 2000 and 2008. This study was approved by the Ethics Committee of Tabriz University of Medical Sciences in Iran (Grant No.: TBZ.93.134) and Iranian Registry of Clinical Trials (IRCT2014122920480N1). Informed consent was obtained from all the parents or guardians before including the children in the study.

The study was carried out during six months from May 2014 to November 2014 in the Department of Pediatric Dentistry, Faculty of Dentistry, Tabriz University of Medical Sciences. The sample size was calculated at 56 subjects (28 in each group) by considering *α*=0.05, a study power of 80% and a success rate of 88% for Articaine and 71% for Lidocaine [19]. All the patients were matched in relation to age and sex. The samples were selected randomly and were randomly assigned to two equal groups using the Randlist software program. The inclusion criteria consisted of the following: Patients with a deciduous maxillary second molar tooth requiring vital pulp therapy, no history of infection, abscess and fistula in the tooth in question, subjects in the early mixed dentition period (6‒9 years of age), absence of internal and external resorption of the root of the tooth, patients in the Frankle category of 3 or 4 in relation to cooperation, no systemic condition in the patients, signing an inflamed consent form by the parents and no history of bedtime and/or spontaneous toothache. The exclusion criteria consisted of the following: Patients with no cooperation, teeth with necrotic pulps, and teeth with hyperemic pulp after the pulp exposure.

**Figure 1:**
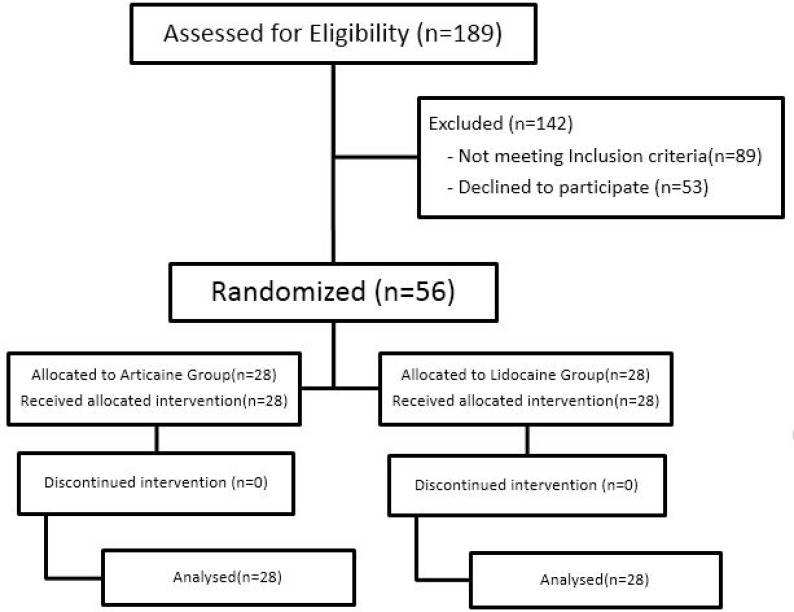
Flow diagram of study patients

Subjects whose maxillary deciduous second molar teeth required vital pulp therapy were randomly divided into two groups. In group 1, one mL of 4% Articaine containing 1:100000 concentration of epinephrine (Artinibsa, Inibsa, Barcelona, Spain) was infiltrated buccally. The needle was inserted into the mucobuccal fold and was advanced to a depth to approach the apices of the buccal roots of the teeth in question. The needle’s bevel was oriented toward the bone and the periosteum and 1 mL of the anesthetic agent was injected [9, 20].

In group 2, one mL of 2% Lidocaine containing 1:80000 epinephrine (DaruPakhsh, Tehran, Iran) was injected in a PSA nerve block technique. The needle was inserted at the height of the mucobuccal fold superior and distal to the distobuccal root of the last molar in the dental arch. Then the cheek was retracted to stretch the mucobuccal fold. The needle bevel was oriented toward the bone and the needle was inserted up to the height of the mucobuccal fold over the last molar. The needle was advanced slowly upward at a 45^°^ angle relative to the occlusal plane, inward toward the midline at a 45^°^ angle relative to the occlusal plane and backward at a 45^°^ angle relative to the long axis of the molar in question up to a depth of 10-14 mm. After aspiration, 1 mL of the anesthetic was injected and several aspirations were carried out during injection [20].

All the injections were carried out by one pedodontists in 45 sec with the use of standard dental syringe with a 27-guage needle measuring 20 mm in length (C-K ject, CK Dental, Kor-Kyungji-do, Korea). 

After 10 min, the caries was removed and access cavity preparation was instituted. The patients were asked to report the presence or absence of pain during the procedure [21].

Therefore, the existence of pain is measured by the patient's self-report. A checklist was used to collect data. It should be pointed out that before injection procedures, the anesthetic agents were prepared by an operator who was not involved in the study procedures and coded in identical syringes. The codes were broken only when data were analyzed; therefore, neither the researcher nor the operator injecting the anesthetic agents, nor were the patients aware of the subjects’ groupings (a double-blind study).

***Statistical analysis***

Data were analyzed with descriptive statistical methods (frequencies, percentages) using SPSS (Statistical Package for Social Science, SPSS, version 17.0, SPSS, Chicago, IL, USA). The *chi*-squared test was used to compare pain frequencies between the two groups. Statistical significance level was set at 0.05.

## Results

**Figure 2:**
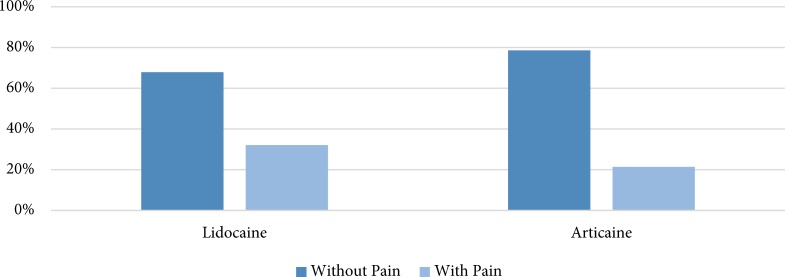
The frequency (%) of cases with and without pain in both groups

A total of 56 children, aged 6‒9 years (7.2±0.6) from both genders were evaluated in the present study. The subjects were candidates for vital pulp therapy of maxillary deciduous second molars, who were randomly assigned to two equal groups. All the subjects completed the study and none was excluded from the study (Figure 1). In the Articaine group, 6 (21.4%) subjects reported pain and 22 (78.6%) reported no pain. In the Lidocaine group, 9 (32.1%) subjects reported pain and 19 (67.9%) reported no pain (Figure 2). However, the *chi*-squared test revealed no significant differences between these two groups (*P*=0.54). No specific complications were seen in any patient in the two groups.

## Discussion

The preset study compared the efficacy of buccal infiltrations of 4% Articaine with that of PSA technique with the use of 2% Lidocaine in the anesthesia of deciduous maxillary second molars in children 6-9 years of age. The results showed that infiltration of Articaine resulted in more effective aesthesia compared to Lidocaine in the PSA technique; however, there were no significant differences between the two groups. Therefore, it can be concluded under the limitations of the present study that buccal infiltration of Articaine in children during the early mixed dentition period in the area of maxillary deciduous molars can be an alternative for the PSA technique with Lidocaine. In comparison to Lidocaine, Articaine has an ester group and a thiophene group which increase its solubility in lipids. On the other hand, considering the 1.5 higher potency of Articaine compared to Lidocaine and its high capacity to penetrate into bone [16, 21, 22]. In a similar study, Arali *et al.* [23] evaluated 40 children aged 5-8 year in relation to the success rates of IANB technique with 2% Lidocaine containing 1:100000 concentration of epinephrine and buccal infiltration of 4% Articaine with a concentration of 1:100000 of epinephrine in the treatment of mandibular deciduous molars with irreversible pulpitis. They suggested that the buccal infiltration of Articaine can be an alternative for the IANB technique with Lidocaine because with the infiltration technique complications such as lip biting decreases, which is common with the IANB technique.

Monteiro *et al.* [24] evaluated 50 adult patients with irreversible pulpitis of mandibular first molars in a double-blind clinical trial. In this study, 20 patients received 2% Lidocaine with 1:100000 epinephrine in the IANB technique and 30 patients received 4% Articaine with 1:100000 epinephrine in a buccal infiltration technique. Evaluation of patients’ pain with an electric pulp tester showed that the success rate of Articaine was significantly higher than that of Lidocaine. In another study, Hosseini *et al.* [25] evaluated 47 adult patients with irreversible symptomatic pulpitis in maxillary molars. In that study a group of patients received buccal infiltration of 2% Lidocaine containing 1:80000 concentration of epinephrine and one group received buccal infiltration of 4% Articaine containing 1:100000 concentration of epinephrine. The success rates of the anesthetic techniques were evaluated with cold test and visual analog scale (VAS). Despite the higher success rate of anesthesia in the Articaine group (66.6%) compared to that in the Lidocaine group (56.5%), the difference was not statistically significant.

Katyal [26] carried out a meta-analysis and reported that the success of the anesthetic technique with Articaine in adults and in children over 4 year of age was higher than that of Lidocaine. Also they concluded that Articaine had higher post-injection pain compared to Lidocaine, but pain scores was negligible clinically. Tortamano *et al.* [27] showed that the initiation of anesthesia and its duration in the inferior alveolar nerve block with 4% Articaine containing 1:100000 concentration of epinephrine was higher than that of 2% Lidocaine containing 1:100000 epinephrine.

Arrow [21] carried out a study on 57 adolescent 11-13 years of age to evaluate the success of Lidocaine with 1:80000 concentration of epinephrine and 4% Articaine in the buccal infiltration technique for restorative procedures of posterior mandibular teeth. Although the success rate of Articaine was higher than that of Lidocaine (71% *vs.* 64%), the difference was not statistically significant.

Bartlett *et al.* [28] carried out a review on the success of IANB anesthetic technique with 2% Lidocaine compared to the buccal infiltration of 4% Articaine and reported that the success rates of Lidocaine and Articaine were 55.6-69.2% and 65.4-70.4%, respectively, concluding that the success of infiltration of Articaine was almost similar to that of IANB technique with Lidocaine. 

The present study, showed for the first time that infiltration of Articaine resulted in more effective anesthesia compared to Lidocaine in the PSA technique; however, there were no significant differences between the two groups. 

In the previous studies, several reasons have been described for the failure of buccal infiltration injections in maxillary molars such as a longer root length, root divergence, pulp inflammation [29]. One of the limitations of the current study was the lack of consideration of root length and divergence. So it is suggested that further studies be carried out with larger sample sizes, with the use of different concentrations of anesthetic agents by considering of the root length and divergence and use of different standard techniques for the evaluation of anesthesia success (cold test, electric pulp tester, *etc*).

## Conclusion

Under the limitations of the present study, there was no significant differences between the results of Articaine buccal infiltration and Lidocaine PSA technique in children during the early mixed dentition period in the maxillary deciduous second molar area, so Articaine buccal infiltration can be used as a substitute for the PSA technique.

## References

[1] Ileri Z, Baka ZM, Akin M, Apiliogullari S, Basciftci FA (2016). Effect of menstrual cycle on orthodontic pain perception. J Orofac Orthop.

[2] Firestone AR, Scheurer PA, Bürgin WB (1999). Patients' anticipation of pain and pain-related side effects, and their perception of pain as a result of orthodontic treatment with fixed appliances. Eur J Orthod.

[3] Appukuttan DP, Tadepalli A, Cholan PK, Subramanian S, Vinayagavel M (2013). Prevalence of dental anxiety among patients attending a dental educational institution in Chennai, India--a questionnaire based study. Oral Health Dent Manag.

[4] Parirokh M, P VA (2014). Various strategies for pain-free root canal treatment. Iran Endod J.

[5] Becker DE, Reed KL (2012). Local anesthetics: review of pharmacological considerations. Anesth Prog.

[6] Cepeda MS, Tzortzopoulou A, Thackrey M, Hudcova J, Arora Gandhi P, Schumann R (2012). Cochrane Review: Adjusting the pH of lidocaine for reducing pain on injection. Cochrane Database Syst Rev.

[7] Clark TM, Yagiela JA (2010). Advanced techniques and armamentarium for dental local anesthesia. Dental clinics of North America.

[8] Padhye M, Gupta S, Chandiramani G, Bali R (2011). PSA block for maxillary molar's anesthesia-an obsolete technique?. Oral Surg Oral Med Oral Pathol Oral Radiol Endod.

[9] Gusi N, Raimundo A, Leal A (2006). Low-frequency vibratory exercise reduces the risk of bone fracture more than walking: a randomized controlled trial. BMC musculoskeletal disorders.

[10] Freuen ND, Feil BA, Norton NS (2007). The clinical anatomy of complications observed in a posterior superior alveolar nerve block. The FASEB Journal.

[11] Chisci G, Chisci C, Chisci V, Chisci E (2013). Ocular complications after posterior superior alveolar nerve block: a case of trochlear nerve palsy. Int J Oral Maxillofac Surg.

[12] Penarrocha-Diago M, Sanchis-Bielsa J (2000). Ophthalmologic complications after intraoral local anesthesia with articaine. Oral Surg Oral Med Oral Pathol Oral Radiol Endod.

[13] Leith R, Lynch K, O'Connell AC (2012). Articaine use in children: a review. Eur Arch Paediatr Dent.

[14] Yilmaz Y, Eyuboglu O, Keles S (2011). Comparison of the efficacy of articaine and prilocaine local anaesthesia for pulpotomy of maxillary and mandibular primary molars. Eur J Paediatr Dent.

[15] Srinivasan N, Kavitha M, Loganathan CS, Padmini G (2009). Comparison of anesthetic efficacy of 4% articaine and 2% lidocaine for maxillary buccal infiltration in patients with irreversible pulpitis. Oral Surg Oral Med Oral Pathol Oral Radiol Endod.

[16] Mittal M, Sharma S, Kumar A, Chopra R, Srivastava D (2015). Comparison of Anesthetic Efficacy of Articaine and Lidocaine During Primary Maxillary Molar Extractions in Children. Pediatr Dent.

[17] Arrow P (2012). A comparison of articaine 4% and lignocaine 2% in block and infiltration analgesia in children. Aust Dent J.

[18] Kanaa MD, Whitworth JM, Meechan JG (2012). A comparison of the efficacy of 4% articaine with 1:100,000 epinephrine and 2% lidocaine with 1:80,000 epinephrine in achieving pulpal anesthesia in maxillary teeth with irreversible pulpitis. J Endod.

[19] Haase A, Reader A, Nusstein J, Beck M, Drum M (2008). Comparing anesthetic efficacy of articaine versus lidocaine as a supplemental buccal infiltration of the mandibular first molar after an inferior alveolar nerve block. J Am Dent Assoc.

[20] Schwartz S (2012). Local anesthesia in pediatric dentistry. Revised January.

[21] Arrow P (2012). A comparison of articaine 4% and lignocaine 2% in block and infiltration analgesia in children. Aust Dent J.

[22] Srinivasan N, Kavitha M, Loganathan CS, Padmini G (2009). Comparison of anesthetic efficacy of 4% articaine and 2% lidocaine for maxillary buccal infiltration in patients with irreversible pulpitis. Oral Surgery, Oral Medicine, Oral Pathology, Oral Radiology, and Endodontology.

[23] Arali V, Mytri P (2015). Anaesthetic Efficacy of 4% Articaine Mandibular Buccal Infiltration Compared To 2% Lignocaine Inferior Alveolar Nerve Block in Children with Irreversible Pulpitis. J Clin Diagn Res.

[24] Monteiro M, Groppo F, Haiter‐Neto F, Volpato M, Almeida J (2015). 4% articaine buccal infiltration versus 2% lidocaine inferior alveolar nerve block for emergency root canal treatment in mandibular molars with irreversible pulpits: a randomized clinical study. Int Endod J.

[25] Hosseini HR, Parirokh M, Nakhaee N, Abbott PV, Samani S (2016). Efficacy of Articaine and Lidocaine for Buccal Infiltration of First Maxillary Molars with Symptomatic Irreversible Pulpitis: A Randomized Double-blinded Clinical Trial. Iran Endod J.

[26] Katyal V (2010). The efficacy and safety of articaine versus lignocaine in dental treatments: a meta-analysis. J Dent.

[27] Tortamano IP, Siviero M, Lee S, Sampaio RM, Simone JL, Rocha RG (2013). Onset and duration period of pulpal anesthesia of articaine and lidocaine in inferior alveolar nerve block. Braz Dent J.

[28] Bartlett G, Mansoor J (2016). Articaine buccal infiltration vs lidocaine inferior dental block–a review of the literature. British dental journal.

[29] Askari EM, Parirokh M, Nakhaee N, Hosseini HR, Abbott PV (2016). The Effect of Maxillary First Molar Root Length on the Success Rate of Buccal Infiltration Anesthesia. J Endod.

